# Remote dielectric sensing for pulmonary congestion assessment: current concepts and clinical perspectives

**DOI:** 10.1093/eschf/xvag241

**Published:** 2026-09-18

**Authors:** Teruhiko Imamura

**Affiliations:** The Second Department of Internal Medicine, University of Toyama, 2630 Sugitani, Toyama, Toyama 930-0194, Japan

**Keywords:** ReDS, Heart failure, Congestion, Diuretics

## Abstract

Pulmonary congestion is a major determinant of symptoms, hospitalization, and prognosis in cardiovascular disease, yet its quantitative assessment remains challenging. Remote dielectric sensing is a non-invasive electromagnetic method that provides a quantitative estimate of pulmonary fluid content within ∼1 min. This narrative review summarizes the principles, validation, clinical interpretation, applications, and limitations of remote dielectric sensing. In a validation study of 46 hospitalized patients, including 28 with heart failure, remote dielectric sensing correlated with computed tomography-derived high-attenuation lung area (*r* = 0.65, *P* < .001). In a prospective study of 153 patients with heart failure, remote dielectric sensing correlated with lung ultrasound B-line count (*r* = 0.544); a threshold of 34.5% identified lung ultrasound-defined pulmonary congestion with an area under the curve of 0.748, sensitivity of 73.5%, and specificity of 70.2%. In 133 patients hospitalized with acute heart failure, with a median age of 78 years, 59% men, and a median left ventricular ejection fraction of 57%, an increase in remote dielectric sensing from admission to discharge was associated with all-cause death or heart failure rehospitalisation (adjusted hazard ratio 4.37, 95% confidence interval 1.13–16.81). In a proof-of-concept randomized trial of 100 patients hospitalized with acute decompensated heart failure, the primary endpoint occurred in 2% with remote dielectric sensing-guided care vs 20% with routine care (hazard ratio 0.094). Available evidence supports diagnostic validity and potential prognostic value, but external validation remains limited. Evidence that remote dielectric sensing-guided management improves clinical outcomes remains preliminary. Remote dielectric sensing should be considered a complementary component of multimodality congestion assessment.

## Graphical Abstract

**Graphical Abstract:**
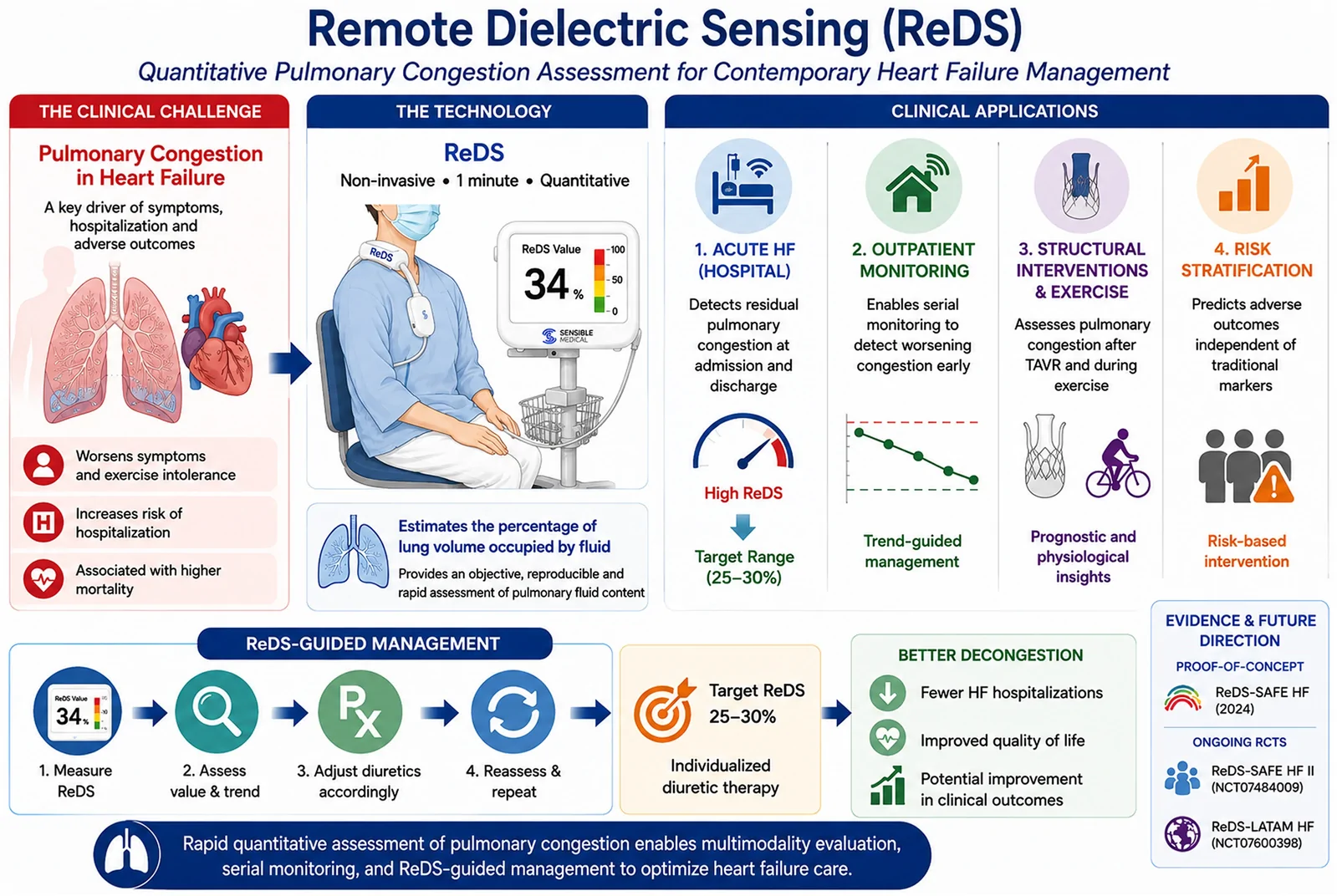
Clinical integration of remote dielectric sensing for pulmonary congestion assessment. Remote dielectric sensing (ReDS) provides a rapid, non-invasive, quantitative estimate of pulmonary fluid content and may complement conventional congestion assessment across inpatient and outpatient settings. Serial ReDS measurements may support longitudinal monitoring, risk stratification, and individualized adjustment of decongestive therapy when interpreted together with clinical findings and other diagnostic modalities. Evidence supporting ReDS-guided management remains preliminary, and adequately powered randomized trials are required to determine its impact on clinical outcomes.

## Introduction

Pulmonary congestion is a central pathophysiological feature of heart failure and a major determinant of symptoms, hospitalization, and adverse clinical outcomes.^1,2^ Pulmonary congestion and elevated intracardiac filling pressure are related but physiologically distinct and may become dissociated in some clinical settings.

Pulmonary congestion develops when elevated left-sided filling pressures increase pulmonary capillary hydrostatic pressure, promoting fluid transudation into the pulmonary interstitium and, when more severe, the alveolar space. Its severity is also influenced by fluid redistribution, vascular permeability, and lymphatic drainage; therefore, pulmonary fluid accumulation does not necessarily parallel systemic volume overload or filling pressure.

Residual pulmonary congestion at discharge is strongly associated with an increased risk of rehospitalization and mortality,^3–6^ whereas excessive decongestion may result in intravascular volume depletion and intolerance to guideline-directed medical therapy.^7–9^ Accurate assessment of pulmonary congestion is therefore essential for optimizing patient management throughout the course of heart failure.^10^

However, quantifying pulmonary congestion remains challenging in routine clinical practice.^2^ Conventional modalities, including physical examination, chest radiography, natriuretic peptides, echocardiography, and right heart catheterization (RHC), each have inherent limitations related to accuracy, invasiveness, operator dependency, or practicality. Consequently, there is an unmet need for a rapid, objective, and non-invasive method to estimate pulmonary fluid accumulation.^11^

**Figure 1:**
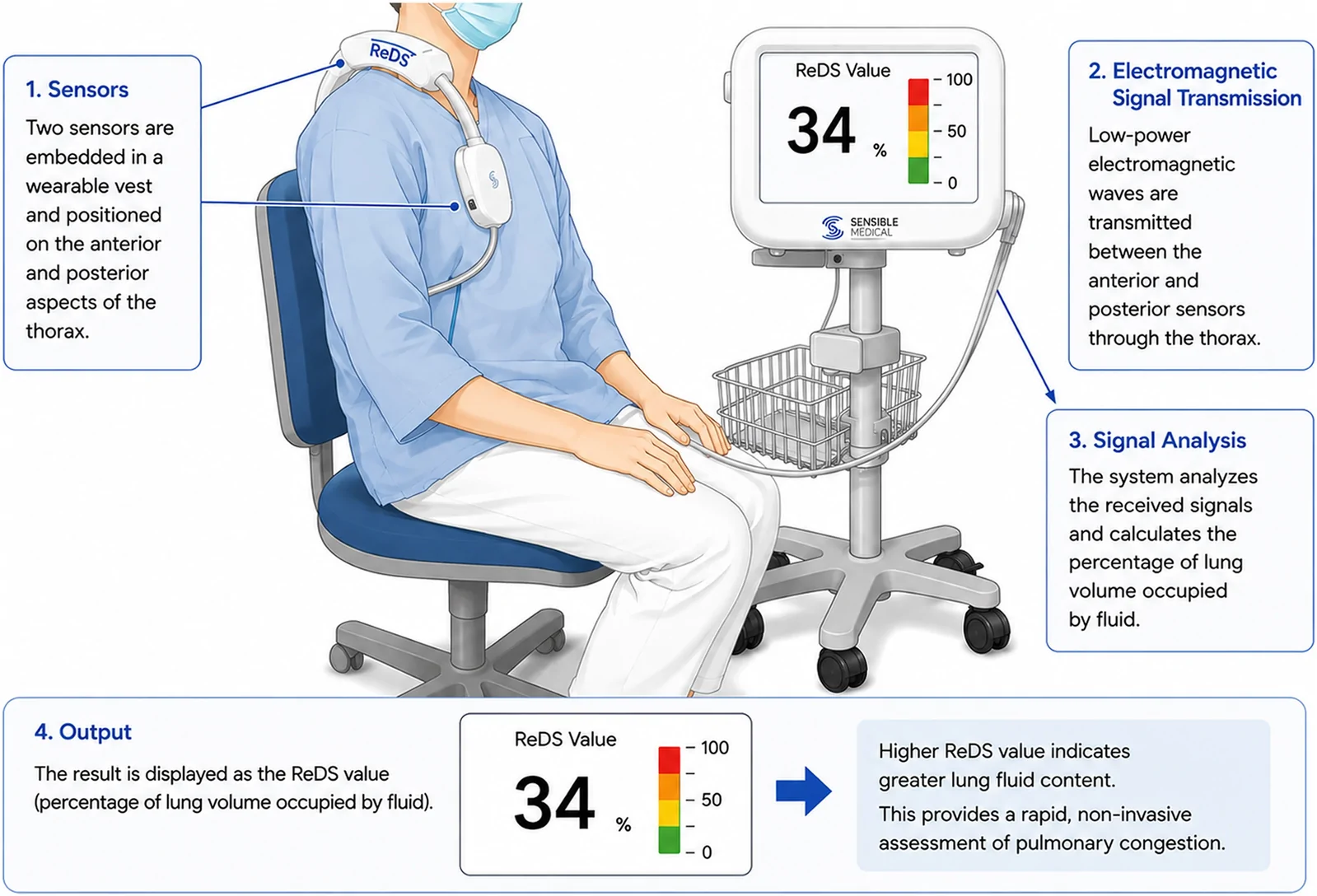
Overview of the ReDS system. The wearable device consists of anterior and posterior sensors that transmit low-power electromagnetic signals through the thorax. The received signals are analyzed to estimate lung fluid content, which is displayed as the ReDS value within ∼1 min

Remote dielectric sensing (ReDS™; Sensible Medical Innovations, Netanya, Israel) is a novel electromagnetic-based technology that quantitatively estimates lung fluid content within ∼1 min (*Figure 1*).^12^ Since its introduction, accumulating evidence has supported its analytical validity and extended its clinical applications across a broad range of cardiovascular conditions. More recently, interest has expanded to ReDS-guided therapeutic interventions.^13^

Given these recent developments, an updated review of the current evidence is warranted. In this review, we summarize the physical principles, analytical performance, and clinical interpretation of ReDS, discuss its integration with conventional congestion assessment modalities, review its current and emerging clinical applications, and highlight its limitations and future perspectives.

## Literature search strategy

A narrative literature search was performed using PubMed/MEDLINE from database inception through July 2026. Search terms included ‘remote dielectric sensing,’ ‘ReDS,’ ‘heart failure,’ ‘pulmonary congestion,’ and ‘lung fluid,’ with additional searches for relevant comparator technologies, including lung ultrasound, bioimpedance, and implantable pulmonary artery pressure monitoring. Randomized and observational studies, meta-analyses, relevant reviews, and major guideline or consensus documents were considered. Studies were selected based on their relevance to the principles, validation, clinical interpretation, applications, limitations, and implementation of ReDS.

## Principles of remote dielectric sensing

**Figure 2:**
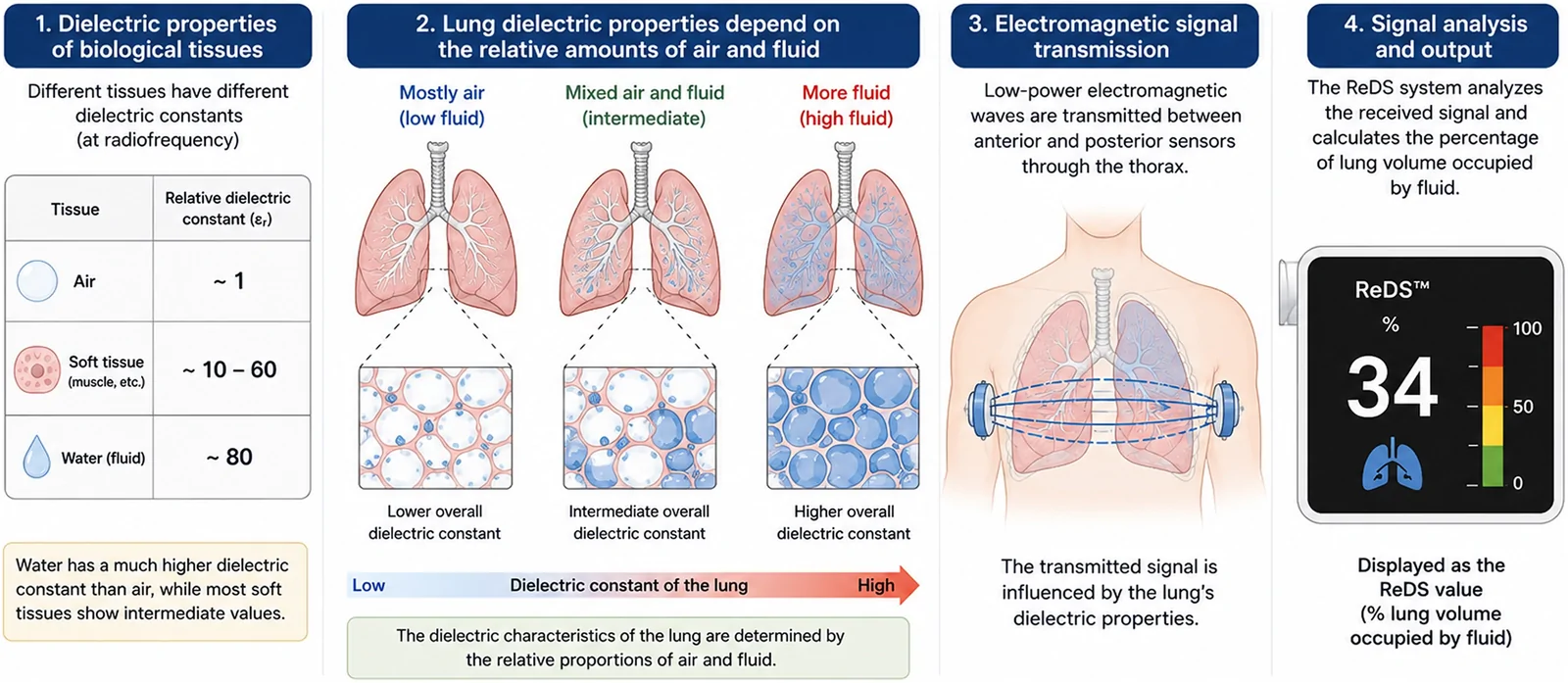
Principles of ReDS. Because water has a substantially higher dielectric constant than air, the dielectric properties of the lung vary according to the relative proportions of air and fluid. By analysing electromagnetic signals transmitted through the thorax, ReDS quantitatively estimates the percentage of lung volume occupied by fluid

ReDS is a non-invasive technology designed to quantify lung fluid content using low-power electromagnetic signals (*Figure 2*).^12^ The system consists of two sensors embedded within a wearable vest, positioned on the anterior and posterior aspects of the thorax. During measurement, electromagnetic waves pass through the thorax, and the received signals are analyzed to estimate lung fluid content.

The principle of ReDS is based on differences in the dielectric properties of biological tissues.^14^ Water has a substantially higher dielectric constant than air, whereas most soft tissues exhibit intermediate values. Consequently, the dielectric characteristics of the lung are largely determined by the relative proportions of air and fluid within the lung.^15^ By analyzing the transmitted electromagnetic signals, the ReDS system estimates the percentage of lung volume occupied by fluid, which is displayed as the ReDS value.

Unlike conventional imaging modalities, ReDS does not generate anatomical images but instead provides a quantitative estimate of lung fluid content.^16^ The measurement is completed within ∼1 min under quiet tidal breathing and requires minimal operator training. ReDS estimates pulmonary fluid rather than intracardiac filling pressure and should not be interpreted as a surrogate for haemodynamic pressure.^17^

## Analytical performance of ReDS

The analytical performance of ReDS has been evaluated against multiple reference standards, including computed tomography (CT), invasive haemodynamic measurements, and other non-invasive modalities for assessing pulmonary congestion (*Table 1*). Overall, available studies support the ability of ReDS to estimate pulmonary fluid content across diverse clinical settings.

**Table 1:** Modalities for assessing pulmonary congestion

Modality	Primary target	Direct assessment of pulmonary congestion	Serial monitoring	Advantages	Limitations	Clinical role
Physical examination	Clinical signs	△	◎	Rapid, inexpensive, universally available	Low sensitivity, interobserver variability	Initial bedside assessment
Chest radiography	Pulmonary oedema, pleural effusion	△	△	Widely available	Low sensitivity for mild congestion; qualitative	Initial evaluation and differential diagnosis
BNP/NT-proBNP	Myocardial wall stress	×	△	Objective biomarker; prognostic value	Influenced by age, obesity, renal dysfunction, atrial fibrillation	Diagnosis, risk stratification, treatment monitoring
Lung ultrasound	Extravascular lung water	◎	◎	High sensitivity; bedside examination	Operator dependent; limited acoustic windows	Detection and serial monitoring of pulmonary congestion
Echocardiography	Cardiac structure and filling pressure	△	×	Comprehensive structural and functional assessment	Indirect assessment of pulmonary congestion; requires expertise	Etiological evaluation
Chest CT	Lung parenchyma	◎	×	Excellent anatomical resolution; differential diagnosis	Radiation exposure; costly; unsuitable for routine follow-up	Selected patients with uncertain diagnosis
Right heart catheterization	Intracardiac filling pressures	×	×	Reference standard for haemodynamic assessment	Invasive; unsuitable for serial monitoring	Advanced heart failure and complex haemodynamic evaluation
Bioimpedance analysis	Systemic fluid status	×	◎	Non-invasive; evaluates whole-body fluid balance	Limited specificity	Adjunctive assessment of systemic congestion
Implantable pulmonary artery pressure monitoring	Pulmonary artery pressure	△	◎	Daily remote haemodynamic monitoring; outcome evidence from randomized trials	Invasive implantation; device and monitoring costs; restricted patient selection	Long-term remote management of selected patients with chronic heart failure
Remote dielectric sensing (ReDS)	Pulmonary fluid content	◎	◎	Rapid, quantitative, non-invasive, reproducible, suitable for repeated measurements	Does not assess cardiac structure; affected by certain pulmonary conditions (e.g. COPD, pleural effusion)	Quantification and longitudinal monitoring

Assessment scale: ◎ = primary/direct assessment; △ = indirect or limited assessment; × = not intended for assessment of pulmonary congestion.

BNP, B-type natriuretic peptide; COPD, chronic obstructive pulmonary disease; CT, computed tomography.

**Figure 3:**
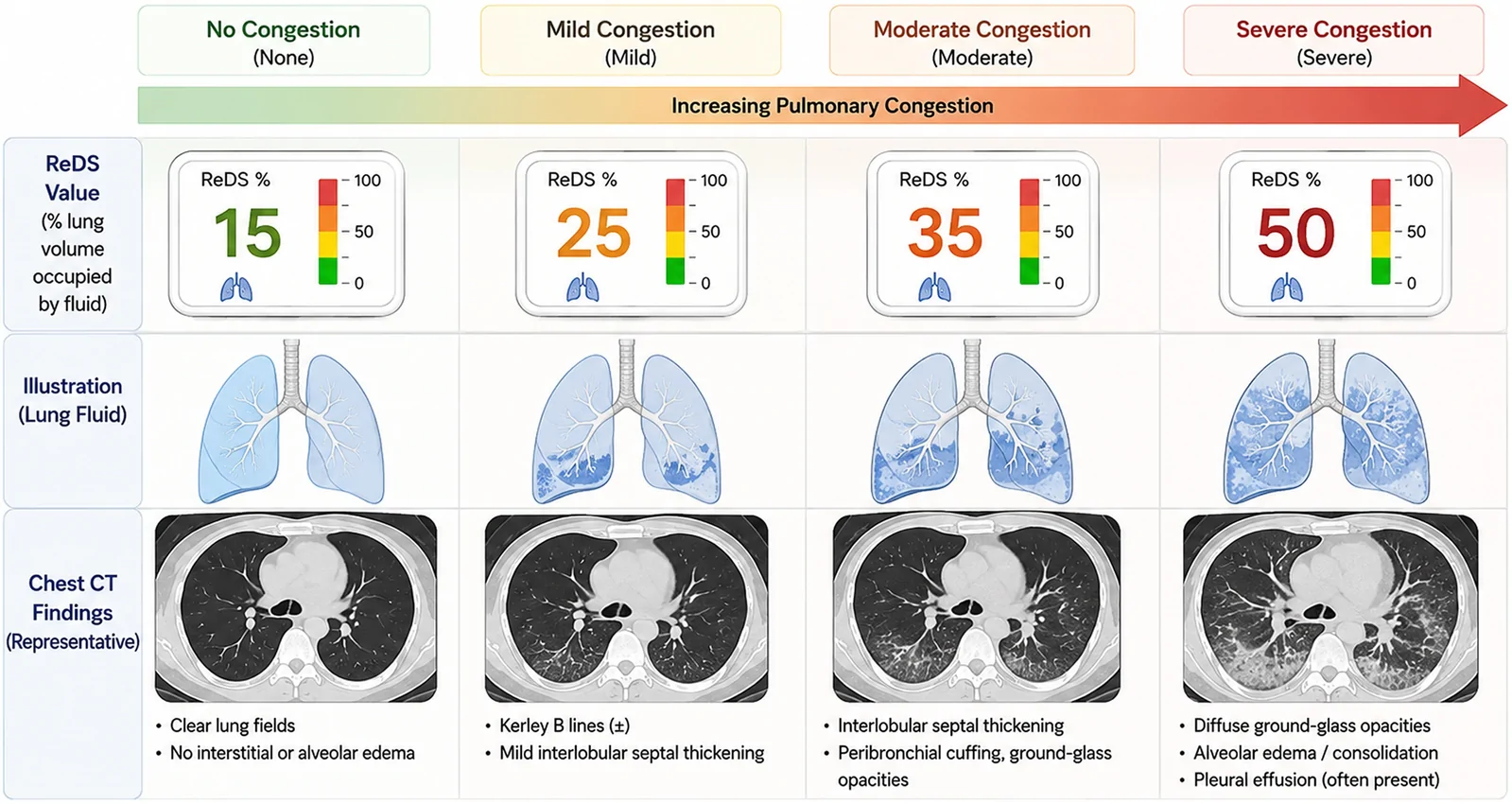
Relationship between pulmonary congestion, illustrative chest CT findings, and ReDS values. Increasing pulmonary fluid accumulation is accompanied by progressive radiographic abnormalities on chest CT and higher ReDS values. ReDS provides a quantitative estimate of pulmonary fluid content across the spectrum of congestion severity. Values are illustrative and do not represent validated severity thresholds

High-resolution CT has served as one of the principal reference standards for validating ReDS. Previous studies demonstrated a moderate-to-strong correlation between ReDS values and CT-derived lung fluid content, supporting an association between ReDS values and pulmonary fluid accumulation (*Figure 3*).^18^ A Japanese validation study also demonstrated a similar relationship in patients with relatively small body size.^14^ Broader external validation remains needed.

Several studies have compared ReDS with invasive haemodynamic measurements.^17,19,20^ ReDS values correlate moderately with pulmonary artery wedge pressure, underscoring that pulmonary fluid and filling pressure are related but not interchangeable. Agreement between ReDS and other routinely used congestion assessment modalities has also been investigated. Moderate correlations have been reported with chest radiography, lung ultrasound, and plasma B-type natriuretic peptide levels, although each modality reflects different aspects of congestion.^21–25^ These comparisons show moderate concordance without establishing interchangeability.

Beyond analytical accuracy, ReDS has demonstrated excellent intraobserver reproducibility and good interobserver reliability.^26^ Together with its rapid acquisition time and minimal operator dependency, these characteristics support the feasibility of ReDS for serial monitoring in routine clinical practice.

## Clinical interpretation of ReDS values

Unlike many conventional congestion assessment tools, ReDS provides a continuous quantitative estimate of lung fluid content rather than a qualitative assessment of congestion (*Figure 3*).^27^ This characteristic allows clinicians to monitor subtle changes in pulmonary fluid volume over time, even in the absence of overt clinical signs or symptoms.^28^ Consequently, interpretation of ReDS values should focus not only on absolute values but also on their temporal changes and the overall clinical context.^29^

The manufacturer-defined normal range of ReDS values is 20% to 35%, with higher values generally indicating greater pulmonary fluid accumulation.^12^ However, pulmonary congestion exists as a continuum rather than a binary phenomenon,^2^ and no single cut-off is universally applicable across all clinical scenarios.^27^ Mild elevations may indicate subclinical congestion in otherwise stable patients,^30^ but their clinical significance may vary according to comorbidities and measurement conditions. Therefore, ReDS values should be interpreted in conjunction with clinical judgment.

**Figure 4:**
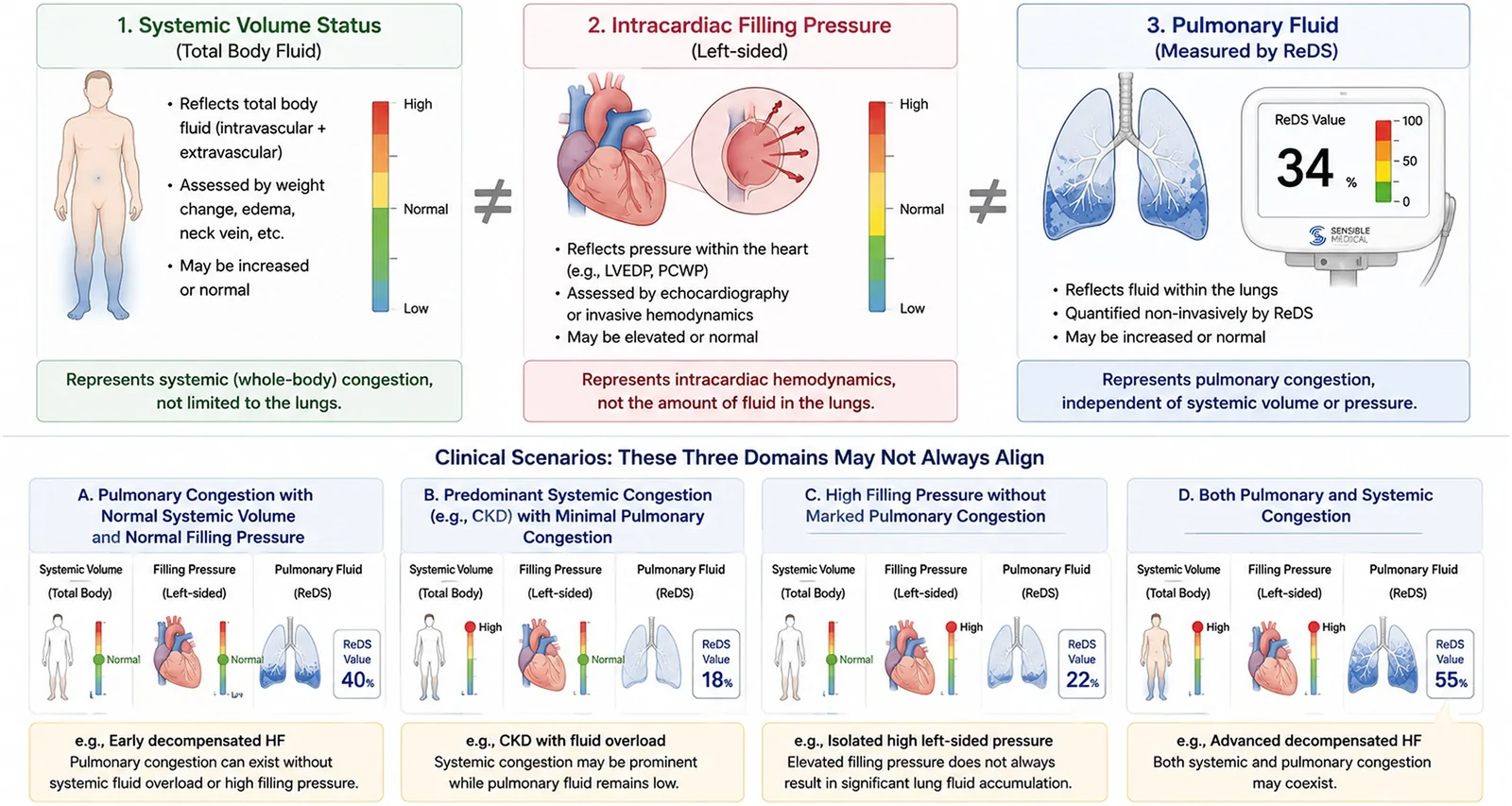
Conceptual relationship among systemic volume status, intracardiac filling pressure, and pulmonary fluid measured by ReDS. These physiological domains sometimes overlap but are not interchangeable. CKD, chronic kidney disease; LVEDP, left ventricular end-diastolic pressure; PCWP, pulmonary capillary wedge pressure

It is also important to recognize that ReDS quantifies pulmonary fluid rather than intracardiac filling pressure or systemic volume status (*Figure 4*).^17^ Although pulmonary congestion frequently accompanies elevated left-sided filling pressures, these physiological processes are not identical.^2^ Likewise, pulmonary and systemic congestion may not always coexist.^7^ Patients with heart failure may exhibit pulmonary congestion despite relatively normal systemic volume status, whereas others, particularly those with chronic kidney disease, may predominantly present with systemic congestion.^31^

Changes in ReDS values over time may better reflect dynamic alterations in pulmonary congestion than isolated measurements and may facilitate assessment of treatment response.^29^

## Integration with other modalities

Assessment of congestion should not rely on a single modality, as each diagnostic tool provides different physiological and clinical information.^2^ Physical examination and chest radiography remain essential first-line tools for congestion assessment because of their wide availability and practicality.^1^ Chest CT provides additional anatomical information when the aetiology of pulmonary abnormalities is uncertain. ReDS may provide additional quantitative information regarding pulmonary fluid burden, particularly when clinical findings are equivocal. Lung ultrasound may further improve diagnostic confidence by identifying the distribution of pulmonary oedema.^32^

Natriuretic peptides reflect myocardial wall stress, whereas echocardiography provides structural and haemodynamic information that cannot be obtained by ReDS alone.^9,33^ Changes in ReDS values should be interpreted together with symptoms, body weight, renal function, and biomarker findings rather than in isolation.

RHC remains the reference standard for invasive haemodynamic assessment. ReDS should not be regarded as a substitute but rather as a non-invasive adjunct for repeated assessment of pulmonary congestion (*Figure 4*).^7^

## ReDS vs lung ultrasound

Lung ultrasound (LUS) is an established point-of-care method for assessing pulmonary congestion through the detection and quantification of B-lines, vertical ultrasound artefacts reflecting increased extravascular lung water. B-lines are sensitive to changes in pulmonary congestion and are useful for diagnosis and serial monitoring, but they are not specific to cardiogenic congestion and may also occur in pulmonary fibrosis or non-cardiogenic pulmonary oedema.^34^

ReDS estimates pulmonary fluid content from the dielectric properties of thoracic tissue. The two techniques therefore assess related but not identical physiological phenomena. Direct comparative evidence remains limited. In a small study of 19 patients with heart failure, a ReDS threshold of ≥35% identified a positive LUS B-profile with a sensitivity of 66.7%, specificity of 87.5%, and negative predictive value of 93.3%. More recently, an independent prospective study of 153 patients demonstrated a moderate correlation between ReDS values and B-line counts (*r* = 0.544); a ReDS threshold of 34.5% identified LUS-defined pulmonary congestion with an area under the curve of 0.748, sensitivity of 73.5%, and specificity of 70.2%. These findings indicate moderate agreement rather than interchangeability between the two modalities.^23^

LUS has important advantages, including widespread availability, bedside anatomical assessment, and the ability to identify the distribution of B-lines and pleural abnormalities. It can also achieve good diagnostic accuracy and reproducibility after appropriate training; in a large emergency-department study, B-line assessment performed by trained residents showed sensitivities of 82.5%–88%, specificities of 75%–84%, and interobserver *κ* values of 0.81–0.85. Nevertheless, image acquisition and interpretation remains operator dependent. ReDS, in contrast, provides a standardized numerical measurement within ∼1 min with minimal operator input, which may facilitate repeated measurements by less specialized personnel.^35^

Both techniques are suitable for serial monitoring, although the evidence base for LUS-guided management is currently more mature, including randomized studies of LUS-guided follow-up, whereas evidence for ReDS-guided treatment remains preliminary.^36^ ReDS may be particularly attractive when rapid, standardized, quantitative longitudinal assessment is desired, whereas LUS may be preferable when anatomical information, regional distribution of congestion, or pleural abnormalities need to be evaluated. Direct comparisons of cost and cost-effectiveness are lacking. LUS may use existing ultrasound equipment but requires operator training and interpretation, whereas ReDS requires a dedicated device but has a shorter learning curve and more standardized acquisition. Accordingly, neither technique should be considered universally superior; their relative value depends on the clinical setting, available expertize, and specific diagnostic question.^22^

## ReDS vs bioimpedance and implantable haemodynamic monitoring

Bioimpedance-based techniques estimate thoracic or systemic fluid status from changes in electrical impedance and can facilitate serial, non-invasive monitoring. However, these measurements provide an indirect estimate of congestion and may be influenced by body composition, electrode configuration, and device-specific algorithms.^37^ In contrast, ReDS is designed to quantitatively estimate pulmonary fluid content within a defined thoracic region. Direct head-to-head comparisons between ReDS and bioimpedance-based techniques remain limited, and their relative incremental value has not been established.

Implantable pulmonary artery pressure monitoring systems, such as CardioMEMS, provide a fundamentally different physiological signal from ReDS. Whereas ReDS estimates pulmonary fluid content, implantable sensors directly measure pulmonary artery pressure and are primarily designed for frequent or daily remote monitoring of selected patients with chronic heart failure. Pulmonary artery pressure may increase before overt clinical congestion develops, allowing earlier therapeutic intervention. Randomized trials of CardioMEMS-guided management have provided a substantially larger evidence base than is currently available for ReDS. The CHAMPION trial demonstrated reductions in heart failure hospitalization,^38^ whereas GUIDE-HF did not significantly reduce the primary composite endpoint in the overall analysis;^39^ the subsequent MONITOR-HF trial demonstrated improvements in health status and reductions in heart failure hospitalization in a European healthcare setting.^40^

In contrast, ReDS is non-invasive and can be used episodically across inpatient and outpatient settings without device implantation. Implantable pulmonary artery pressure monitoring requires an invasive implantation procedure, dedicated remote-monitoring infrastructure, and selection of patients likely to benefit from long-term haemodynamic surveillance. CardioMEMS is currently indicated in the United States for selected patients with NYHA class II or III heart failure with prior heart failure hospitalization and/or elevated natriuretic peptides, whereas the Cordella pulmonary artery sensor is approved for selected patients with NYHA class III heart failure. Costs also differ substantially in structure: implantable systems involve device implantation and longitudinal remote-monitoring resources, whereas ReDS requires dedicated non-invasive equipment but no implanted hardware. Formal cost-effectiveness analyses have been reported for CardioMEMS, whereas robust economic evaluations of ReDS remain limited.^41^ Accordingly, these technologies should be viewed as complementary approaches targeting different stages of the congestion pathway rather than as directly interchangeable monitoring strategies.

## Cost and implementation considerations

Beyond diagnostic performance, implementation depends on acquisition costs, reimbursement, and healthcare resource use. Formal cost-effectiveness data for ReDS remain limited and may vary across healthcare systems. Prospective studies should assess healthcare resource utilization, quality-adjusted life-years, and cost-effectiveness.

## Factors influencing ReDS measurements

**Figure 5:**
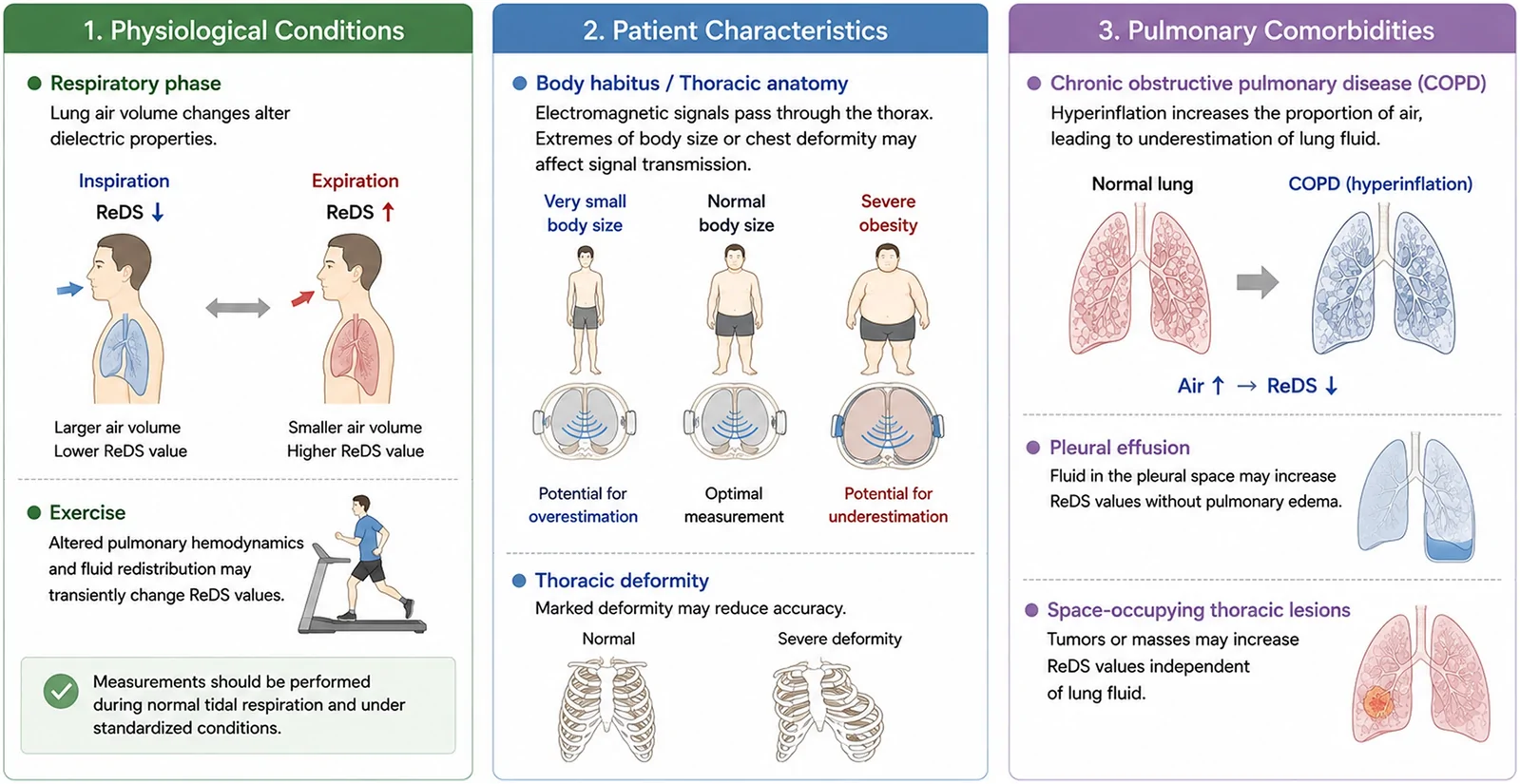
Factors influencing ReDS measurements. Physiological conditions (respiratory phase and exercise), patient characteristics (body habitus and thoracic anatomy), and pulmonary comorbidities (e.g. chronic obstructive pulmonary disease and pleural effusion) may affect ReDS values independent of changes in pulmonary fluid. Standardized measurement conditions and appropriate clinical interpretation are therefore essential

ReDS values may be influenced by physiological and pathological factors independent of changes in total lung water (*Figure 5*). Respiratory phase alters lung air volume and consequently the dielectric properties of the lung.^15^ ReDS values are generally lower during inspiration and higher during expiration than during quiet breathing, supporting the manufacturer's recommendation that measurements should be obtained during normal tidal respiration. Similarly, transient changes associated with exercise may occur due to alterations in pulmonary hemodynamic and fluid redistribution,^42^ suggesting that measurements should be performed under standardized conditions whenever possible.

Patient characteristics may also influence measurement accuracy. Because ReDS relies on electromagnetic signals passing through the thorax, body habitus and thoracic anatomy can affect signal transmission.^18^ Extreme body habitus or marked thoracic deformity may affect measurement accuracy, although supporting data remain limited.^43^

Pulmonary comorbidities may also affect ReDS values. Chronic obstructive pulmonary disease (COPD) may lead to underestimation of lung fluid because hyperinflation increases the proportion of air within the lung.^44^ Conversely, pleural effusion may increase ReDS values without necessarily reflecting pulmonary parenchymal oedema.^45^

Accordingly, ReDS values should be interpreted cautiously in patients with severe COPD, pleural effusion, extreme body habitus, or marked chest wall deformity. Unexpectedly low values in COPD or high values in pleural effusion should prompt confirmation with complementary clinical or imaging findings. In these settings, serial trends under standardized conditions may be more informative than a single absolute ReDS value.

## Clinical application across cardiovascular medicine

ReDS has been studied across several cardiovascular settings as an adjunctive measure of pulmonary fluid status.^27^ In patients hospitalized with acute heart failure, ReDS may facilitate assessment of residual congestion during decongestive therapy.^46^ Serial measurements can complement conventional clinical evaluation and help identify patients with persistent pulmonary congestion despite apparent symptomatic improvement. Declining ReDS values may indicate successful decongestion.

During outpatient follow-up, ReDS may facilitate longitudinal assessment of congestion while supporting optimization of diuretic therapy.^47,48^ Preliminary studies suggest that this approach may support individualized volume management in selected patients.

Beyond heart failure, ReDS has been investigated in patients undergoing structural heart interventions. Following transcatheter aortic valve replacement, residual congestion assessed by ReDS has been associated with adverse clinical outcomes.^49^ ReDS has also been used during exercise testing to characterize dynamic changes in pulmonary congestion.^42^ In heart failure with preserved ejection fraction, exercise may provoke pulmonary congestion despite limited congestion at rest. ReDS may provide a quantitative assessment of these dynamic changes in lung fluid, although evidence for its use during exercise remains limited.

Emerging applications include paediatric cardiovascular disease and haemodialysis.^50,51^ Early studies suggest that ReDS may provide clinically useful information when interpreted in conjunction with disease-specific findings. Serial changes and derived ReDS indices have been associated with subsequent heart failure events, suggesting potential roles in longitudinal monitoring and risk stratification.^29,52^

**Figure 6:**
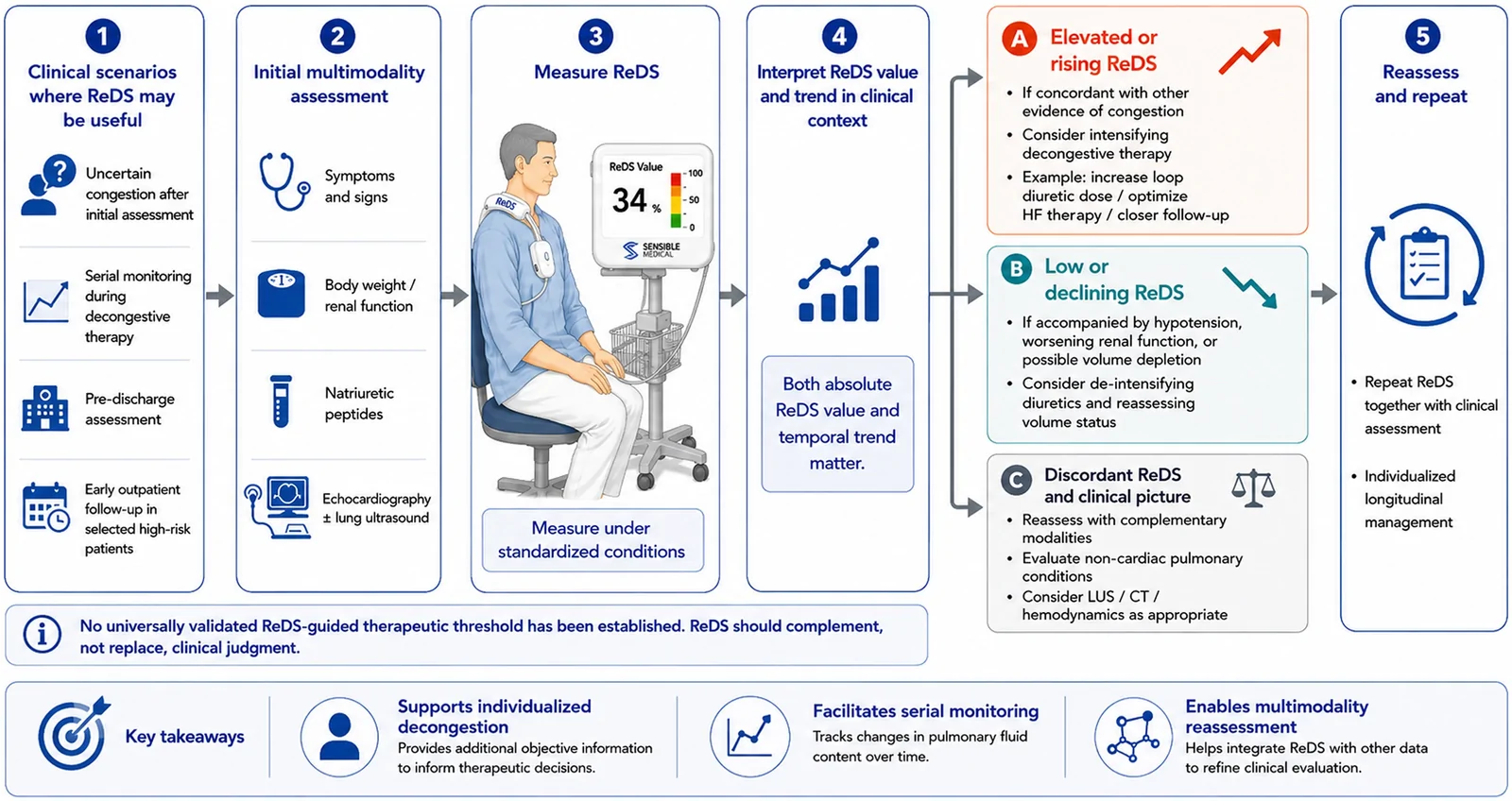
Proposed clinical workflow for integrating ReDS into multimodality congestion assessment. ReDS values and temporal trends should be interpreted together with clinical findings and complementary modalities to inform individualized decongestive management. No universally validated ReDS-guided therapeutic threshold has been established

These observations have prompted interest in ReDS-guided management (*Figure 6*).^47^ ReDS may be particularly useful when pulmonary congestion remains uncertain after initial assessment, during decongestive therapy, before discharge, and during early follow-up in selected high-risk patients. When concordant with other evidence of congestion, elevated or rising ReDS values may support intensification of decongestive therapy, whereas low or declining values in the setting of hypotension, worsening renal function, or other signs of volume depletion may support de-intensification. Discordance between ReDS and the overall clinical picture should prompt reassessment with complementary modalities or evaluation for non-cardiac pulmonary conditions rather than automatic treatment adjustment. ReDS-guided management is most directly relevant to adjustment of loop diuretics. Although sodium-glucose cotransporter two inhibitors may facilitate decongestion in heart failure, no evidence currently supports initiating or titrating these agents according to ReDS values.

The current evidence for ReDS should be interpreted at three distinct levels. First, diagnostic validation studies support its ability to quantitatively assess pulmonary fluid, although external validation remains limited. Second, observational studies suggest that elevated or residual ReDS values are associated with subsequent adverse heart failure outcomes, supporting a potential prognostic role. Third, evidence that ReDS-guided therapeutic interventions improve clinical outcomes remains preliminary. Accordingly, diagnostic validity and prognostic associations should not be interpreted as evidence of therapeutic efficacy, and adequately powered multicenter randomized trials are required before routine ReDS-guided management can be recommended (*Table 2*).

**Table 2:** Representative clinical studies and ongoing trials

Clinical domain	Study	Design/*N*	Population	Follow-up	Primary objective/endpoint	Principal findings	Major limitations
Analytical validation	Amir *et al*.^18^	Cross-sectional validation; *N* = 31	Patients with and without ADHF	—	Validation against chest CT-derived lung fluid	ReDS showed close agreement with CT-derived lung fluid quantification; mean absolute difference was 3.75%.	Small validation cohort; cross-sectional assessment; no clinical outcomes
	Uriel *et al*.^19^	Prospective single-centre validation; *N* = 139	HF patients undergoing RHC	—	Relationship between ReDS and invasive haemodynamics	ReDS was associated with PAWP, supporting a relationship between pulmonary fluid and filling pressure, although the two measures were not interchangeable.	Selected RHC population; single centre; moderate association; no outcome assessment
	Imamura *et al*.^14^	Prospective validation; *N* = 46	Hospitalized patients with and without HF; 28 with HF	—	Validation against CT-derived high-attenuation lung area	ReDS correlated moderately with CT-derived high-attenuation area (*r* = 0.65, *P* < .001).	Small single-centre cohort; measurements after clinical stabilization; limited external validation
Comparative assessment	Huang *et al*.^21^	Prospective single-centre observational study; *N* = 153	Patients with HF	—	Comparison with LUS B-lines	ReDS correlated with B-line count (*r* = 0.544); AUC for LUS-defined congestion was 0.748, with 73.5% sensitivity and 70.2% specificity at 34.5%.	Single centre; LUS used as comparator rather than a gold standard; no outcome assessment
Prognostic assessment	Izumida *et al*.^29^	Prospective single-centre cohort; *N* = 133	Patients hospitalized for acute HF	Median 363 days	Composite of all-cause death or HF rehospitalization	An increase in ReDS from admission to discharge was associated with the primary outcome (adjusted HR 4.37, 95% CI 1.13–16.81).	Single centre; limited number of events; data-derived ReDS ratio threshold; selected population
	Imamura *et al*.^49^	Observational cohort; *N* = 42	Patients undergoing TAVR	Median 316 days	Composite of death or readmission	Higher discharge ReDS was associated with the composite endpoint; a data-derived cut-off of 30% stratified subsequent risk.	Very small single-centre cohort; observational design; data-derived cut-off; no intervention
Novel clinical applications	Imamura *et al*.^42^	Prospective proof-of-concept study; *N* = 13	Chronic HFrEF undergoing CPET	Median 69 days	Dynamic change in lung fluid during exercise	ReDS remained unchanged after CPET in 12 of 13 patients; marked increase occurred in one patient receiving inotropic support.	Very small cohort; HFrEF only; no control group; exploratory study
	Okabe *et al*.^50^	Cross-sectional feasibility study; *N* = 21	paediatric patients with prior Fontan surgery	—	Feasibility and comparison with chest X-ray congestion	ReDS was feasible in all patients and showed a modest correlation with radiographic congestion (r = 0.47).	Small, highly selected Fontan cohort; cross-sectional; no clinical outcomes
	Fujioka *et al*.^51^	Two-case report; *N* = 2	Patients receiving maintenance haemodialysis	Single dialysis session	Feasibility of serial ReDS during fluid removal	ReDS changes differed substantially despite fluid removal, suggesting dissociation between systemic fluid removal and pulmonary fluid changes.	Two cases only; descriptive; no comparator or outcome assessment
ReDS-guided management	Alvarez-Garcia *et al*.^13^ (ReDS-SAFE HF)	Multicenter, single-blind randomized proof-of-concept trial; *N* = 100	Hospitalized ADHF	1 month	Unplanned HF visit, HF rehospitalization, or death	Primary endpoint occurred in 20% vs. 2% with routine vs. ReDS-guided care (HR 0.094), mainly driven by fewer HF readmissions.	Small proof-of-concept trial; short follow-up; few events; not powered for mortality
	ReDS-SAFE HF II (NCT07484009)	Multicenter randomized trial; planned *N* = 1014	Adults hospitalized with HF	30 days	Cardiovascular events after discharge; safety and economic outcomes	Ongoing; no outcome results available.	Ongoing trial; efficacy and cost-effectiveness remain unknown
	ReDS-LATAM HF (NCT07600398)	Pragmatic single-centre randomized trial; planned *N* = 216	Patients hospitalized with acute HF	30 ± 5 days	All-cause death, HF rehospitalization, or unplanned HF visit	Ongoing; no outcome results available.	Ongoing; single centre; no results yet

ADHF, acute decompensated heart failure; AUC, area under the curve; CPET, cardiopulmonary exercise testing; CT, computed tomography; HF, heart failure; HFrEF, heart failure with reduced ejection fraction; LUS, lung ultrasound; PAWP, pulmonary artery wedge pressure; RHC, right heart catheterization; TAVR, transcatheter aortic valve replacement.

## Current limitations

Despite its advantages, ReDS should not be considered a standalone tool for congestion assessment. It quantifies pulmonary fluid content but does not identify the underlying cause of congestion or provide information on cardiac structure, ventricular function, or intracardiac hemodynamics.^27^ Therefore, ReDS should always be interpreted in conjunction with the overall clinical context.

Another important limitation is that ReDS reflects pulmonary congestion rather than overall volume status.^2,7^ Patients with predominant systemic congestion, such as those with peripheral oedema or ascites, may have normal ReDS values, whereas pulmonary abnormalities unrelated to heart failure, such as pleural effusion or COPD, may influence ReDS values. Consequently, an abnormal ReDS value should prompt further clinical evaluation rather than be regarded as a definitive diagnosis.

The evidence base for ReDS remains limited. Most studies are small, observational, single-centre investigations, and several originate from a limited number of research groups, raising concerns about generalizability, publication bias, and external validation. Robust evidence that ReDS-guided management reduces heart failure hospitalization or mortality is lacking.

## Future perspectives

The future role of ReDS within multimodality congestion assessment remains to be defined. Research should focus on standardized monitoring protocols, clinically meaningful intervention thresholds, and disease-specific monitoring strategies.

Integration of serial ReDS measurements into telemonitoring platforms may enable remote detection of worsening pulmonary congestion and facilitate earlier therapeutic intervention. Combining longitudinal ReDS data with symptoms, vital signs, biomarkers, and other remote-monitoring variables using artificial intelligence-based decision support may further improve individualized risk assessment, although prospective validation is required.

The recent proof-of-concept ReDS-SAFE HF trial has provided preliminary evidence supporting the feasibility of ReDS-guided management in acute heart failure.^13^ Ongoing randomized trials, including ReDS-SAFE HF II (NCT07484009) and ReDS-LATAM HF (NCT07600398), are expected to clarify whether ReDS-guided management can improve decongestion and clinical outcomes.

## Conclusions

ReDS is a rapid, non-invasive technology that provides a quantitative estimate of pulmonary fluid content. Available evidence supports its diagnostic validity and suggests potential prognostic value across several cardiovascular settings. However, evidence that ReDS-guided management improves clinical outcomes remains limited. ReDS should be considered a complementary tool within multimodality congestion assessment, while independent multicenter studies and adequately powered randomized trials are needed to define its role in routine practice.

## Declarations

### Data Availability

No data were generated or analysed for this manuscript.
